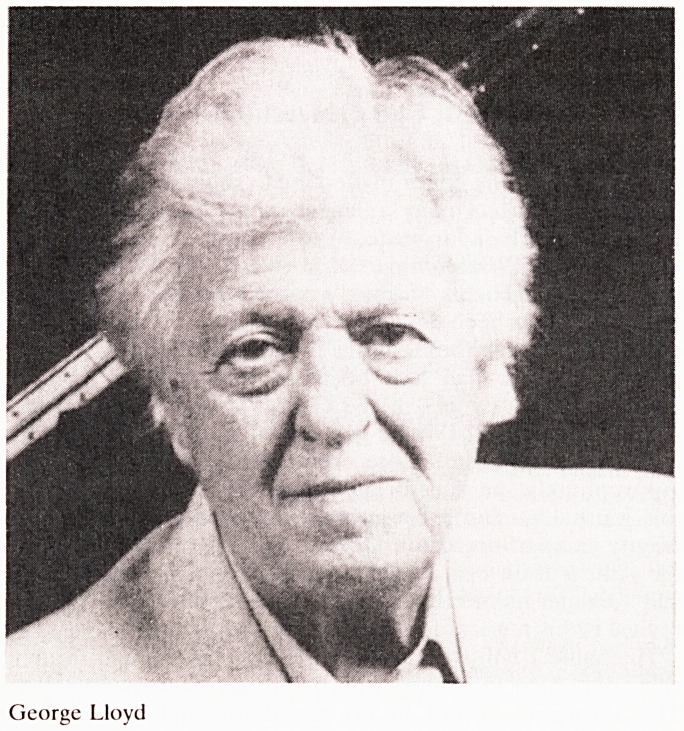# A West Country Composer

**Published:** 1990-06

**Authors:** Alick Dowling

**Affiliations:** General Practitioner (Retd), Bristol


### A West Country Composer

**Alick Dowling, MB, ChB** General Practitioner (Retd), Bristol

During an interval at the Welsh National Opera's Rosenkavalier, the editor invited me to submit a piece to this journal. One meets many colleagues at the opera or concerts, so music must be a favourite, even therapeutic relaxation for many doctors. While immersed in the almost decadent high romanticism of Richard Strauss's music I thought of the music of one who has been described as "almost the only romantic composer left on these shores", and whose most recently premiered work had also been performed by the Welsh National Opera Orchestra and Chorus last November at the Royal Fesival Hall. I invite you therefore to share my interest, an enthusiasm since retirement, for his music. Appropriately, he is associated with the region covered by this journal—a Cornishman born and bred who spent over twenty years of neglect in Dorset, after the pivotal event of the failure of an opera premiered in Bristol in 1951. Yet he still remains unknown to many, despite the extraordinary revival of his musical fortunes during the past dozen years.

His name I will withhold for the moment though further clues may help to identify him before his name is revealed. The surprising upturn in his reputation and his growing popularity are the more remarkable after the neglect of his music, with the exception of the disastrous 1951 production of an opera, for nearly forty years. Intriguing though his life story is, it is the quality of his music that makes it worthwhile for it to be introduced to a larger audience; a recommendation would be of little use unless his music was readily available. Fortunately much of his output is now recorded, mostly on compact disc, having bypassed 78s and even to a large extent LP. His story falls into three well defined parts, early success, musical neglect since the war (when he was injured), and a heart-warming late rediscovery beginning in his mid sixties.

Born in Cornwall in 1913—and thus an exact contemporary of Benjamin Britten—his early musical successes were remarkable by any standards. He began playing the violin at five, started composing at ten, later studied the violin under Albert Sammons at the same time as Yehudi Menuhin, and conducted the Bournemouth Municipal Orchestra in his first Symphony, composed at 19. A year later he conducted the Eastbourne Municipal Orchestra in his second. It was 53 years before it was next performed, in a new revision again under the composer's baton. At 21 his first opera, *Iernin* with a libretto supplied by his father, on a Cornish legend was given at Penzance. By a lucky chance, the music critic of The Times, Frank Howes there on holiday, wrote an enthusiastic notice. His father on the strength of this, formed an Opera Company and booked the Lyceum Theatre in London the following year where *Iernin* ran for a record breaking run, ended only by the heat wave of that 1935 summer which closed so many theatres. Most of the major figures of the British musical establishment came, including Beecham, Ireland, Walton and Vaughan-Williams; the latter, then 63 was heard to observe: "It's not fair; I've spent all my life trying to write operas, and here's this young lad does it first time". As a result of this success, and on the recommendation of John Ireland, his third symphony was played by the BBC Symphony Orchestra with the composer conducting.

In 1938 a second opera, *The Serf*, again with a paternal libretto was staged at Covent Garden under Albert Coates, and toured to Liverpool and Glasgow. It was described as "the most successful thing that has appeared on the native stage since Rutland Boughton's *The Immortal Hour*", but further musical activity was cut short by the 1939 war.

The young composer served in the Royal Marines, most of his service being in the Arctic on convoy duty. After many engagements his ship (HMS Trinidad) was sunk and he was severely shell-shocked. Invalided out in 1942 he was unable to do any work for over three years. In 1945 he went to Switzerland with his Swiss-born wife whom he had married in 1936, to continue his convalescence. While there he wrote his Fourth and Fifth Symphonies, both substantial works. In 1948 he returned to an England in the grip of austerity where he found there was no demand any longer for his music; the two new symphonies remained unperformed. However he and his father were commissioned by the Arts Council to provide a new opera for the 1951 Festival of Britain. There were three opera companies at that time in this country. Commissions were given to Vaughan Williams who provided *Pilgrim's Progress* for Covent Garden, Benjamin Britten who wrote *Billy Budd* for Sadlers Wells and our subject wrote *John Socman* for the Carl Rosa. It was premiered in Bristol and had a number of performances in 1951. Circumstances of that production, quarrels galore, the producer not speaking to the conductor, combined with a recurrence of ill-health partly due to the pressure of producing the opera on schedule—the only one of the three so to appear—so distressed the composer, that he retired from the musical scene. He settled with his wife in Dorset, where for more than twenty years they survived by growing mushrooms and carnations.

As his health improved he continued to compose around the demands of his business; during this time of exclusion by the musical establishment, he wrote four more Symphonies, four Piano Concertos, two Violin Concertos, and a number of piano pieces including a substantial work for John Ogdon in 1966. In 1973 he sold the business and moved to London to resume composing full time. It was not until 1977 that his music began to emerge again. The BBC Northern Symphony Orchestra (later renamed the BBC Philharmonic) gave the first performance of his Eighth Symphony under Edward Downes, who had begun his career as repetiteur at the ill-fated production of *John Socman* in 1951. He had now reached a position when he could introduce music by a composer whose opera he had known well 26 years previously. The positive response from the public induced more performances of other works that were waiting to be heard. Edward Downes continued to be the sole champion to introduce other Symphonies, including the premiere of his Fourth at the Cheltenham Festival in 1981—it had waited 35 years before it saw the light of day, and was highly acclaimed. It is unusual for a substantial modern piece (it lasts 65 minutes) to be so received, with a standing ovation. The composer has described how he was trying to recreate the feelings he had experienced in the Arctic when on convoy duty with the Royal Navy. Edward Downes also gave a memorable first performance of his Ninth Symphony at a Prom the same year.

In 1984 after nearly fifty years, the composer returned to the rostrum to conduct the first performance of his Fourth Piano Concerto with Kathryn Stott and the LSO; he has since conducted many more premieres, including in 1986 his Eleventh Symphony in the USA with the Albany Symphony Orchestra, of which he is now principal guest conductor, and who commissioned it. Another substantial work, it was also received with acclamation at its first performance in this country in Bradford under the composer's baton. A Twelfth Symphony, also commissioned by the Albany Symphony Orchestra, is due to have its first UK performance this year at the Three Choirs Festival in Worcester Cathedral on Friday August 24th. It was rapturously received at its premiere at Albany, New York State on March 30th this year, when the composer conducted it at a concert which included his First Symphony, receiving its first performance since before the war. A CD of the two symphonies—separated by nearly 60 years —is promised for July 1990. The most recent premiere was of a Choral Pageant *The Vigil of Venus* by the W.N.O. though it had been written ten years earlier.

What then is his music like? An impossible question to answer satisfactorily. It is variously described as neo-romantic, melodic, traditional in style yet clearly modern, extrovert, cheerful and instantly approachable. It should be heard, not described; we can turn to recordings; available are: all his eleven symphonies except Nos 1 and 3, two piano concertos as well as a number of piano pieces, and some music for brass. Of the pre-war compositions there is only the 2nd Symphony and no opera except the *John Socman* Overture. There is no vocal music but *The Vigil of Venus*, recorded by the W.N.O., should appear this year.

But why one asks has his music been kept so long from a public ready to welcome it. There is a clear impression that there is opposition to his style from much of the musical establishment. Though the BBC have performed a great service in his initial rehabilitation and commissioned the Tenth Symphony for brass, performances on radio remain sparse. A suggestion that he should be considered for the composer of the week slot was brushed aside. His opera *Iernin* seemed to some at the BBC at its revival under the composer's baton in 1985 to be "impossibly naive"; others would see it as endearingly innocent. Ronald Stevenson, the Scottish composer has attributed his enduring Celtic innocence to the fact that early childhood illness prevented him from attending school until he was twelve. In these circumstances his father, a flute player and lover of Italian opera, must have been a significant influence; the composer has acknowledged Verdi and Berlioz as mentors. It appears that the ready accessibility of his music has ironically been regarded as a black mark against it. On the subject of naivety the composer wrote in programme notes for the first performance in 1980 of his Sixth Symphony, composed in 1956 five years after his withdrawal from the musical scene:

> "Like my first three symphonies, the Sixth is comparatively short. The aim is to be concise, bright and lively with a minimum of development. After I had finished the score, I showed it to one or two people who condemned it as having no contemporary significance; I suppose they thought it was not serious enough for a symphony. Perhaps I was a little naive, but I believed it should once again be possible to say something true, that grew up out of the main tradition of Western music. It also had to have plenty of tunes, because I like music that sings and moves along in a flowing way."

To have music excluded because it has no contemporary significance is precisely what is wrong with our musical and other artistic establishments. It is only in this century, and particularly in the second half, that the heresy has gained acceptance that music, and indeed other arts must be difficult to have any validity. We are bombarded with incomprehensible poetry, ugly sculpture, modern art which bears little resemblance to anything recognisable and music that is cacophonous, discordant and tuneless. The rare gift of melody is derided. The fact that his music is immediatly attractive and worse still actually liked by ordinary listeners would seem to be sufficient reason for critical dismissal.

One is reminded of the sustained attempt some years ago after the death of Sibelius to have his music excluded from the repertoire, orchestrated by those critics who feel they have the right to tell us what we should hear. The public who knew and loved Sibelius, were too numerous to be bamboozled; record companies continued to produce recordings, concert promoters persisted in programming his works and the attempt fizzled out.

There is an analogy with our composer. A correspondent in The Gramophone eager to try new music, has concluded that in the contemporary field his music is like a life raft to a drowning man. Many would echo this wish for intelligent, beautifully orchestrated, well constructed music, even if its 'contemporary significance' is suspect. When performed, its popularity creates a desire for recordings which record companies are willing to satisfy; they would not do so if there were no demand. Those who have yet to experience his music have a treat in store. The name of the composer is of course George Lloyd.

George Lloyd

**DISCOGRAPHY**

**Symphonies**

2 (1933, revised 1982) and 9 (1969) (1986, Conifer)
4 (1946) Albany S.O (G. Lloyd) (1989, Albany), Philharmonia Orchestra (E. Downes) (1984, Lyrita)
5 (1947–8) BBC Phil Orch (G. Lloyd) (1990, Albany), Philharmonia Orchestra (E. Downes) (1982, Lyrita)
6 (1956) & 10 (1981) for Brass and Overture *John Socman*, BBC Phil Orch and Brass (G. Lloyd) (Albany)
7 (1957) BBC Phil Orch (G. Lloyd) (1987, Conifer)
8 (1961–5) Philharmonia Orchestra (E. Downes) (1982, Lyrita)
9 (1969) coupled with No 2, see above (1986, Conifer)
10 for Brass (1981) "November Journeys" coupled with No 6, see above (Albany). London Collegiate Brass/J. Stobart (1987, Trax) (coupled W. Josephs, Concerto for Brass)
11 (1985) Albany S.O. (Lloyd) (1987, Conifer)

**Piano Concertos**

No 3 (1968) Kathryn Stott/BBC Phil Orch (G. Lloyd) (Albany)
No 4 (1970, orchestrated 1983) and other solos, Kathryn Stott/LSO (G. Lloyd)

Lily leaf and Grasshopper (1972), Transformation of That Naked Ape (1972) (1984, Albany)

An African Shrine (1964) and other Piano solos: The Road through Samarkand (1972), St Antony and the Bogside Beggar, The Aggressive Fishes, Intercom Baby. Martin Roscoe (Piano) (1989, Albany)

Royal Parks (1984) Black Dyke Mills Band/Parkes (CHAN)

The Forest of Arden—Symphonic Suite (1987) City of London Wind Ensemble/Brand (1989, LDR)

Wantage Bells (Betjeman) English Songs Recital, Elizabeth Harwood/J. Constable Recital (1984, Conifer)

Lament, Air and Dance, Sonata (Violin and Piano) Tasmin Little and Martin Roscoe (1990, Albany)

## Figures and Tables

**Figure f1:**